# Family Connections: The Impact of an Education Program for Carers of Individuals With Borderline Personality Disorder in Italian Mental Health Services

**DOI:** 10.1111/famp.13098

**Published:** 2025-01-28

**Authors:** Mariangela Lanfredi, Serena Meloni, Clarissa Ferrari, Alan E. Fruzzetti, Andrea Geviti, Ambra Macis, Giovanna Vanni, Giampaolo Perna, Giuseppina Diaferia, Maddalena Pinti, Giorgia Occhialini, Maria Elena Ridolfi, Roberta Rossi

**Affiliations:** ^1^ Unit of Psychiatry IRCCS Istituto Centro San Giovanni di Dio Fatebenefratelli Brescia Italy; ^2^ Research and Clinical Trials Office Fondazione Poliambulanza Istituto Ospedaliero Brescia Italy; ^3^ McLean Hospital and Harvard Medical School Boston Massachusetts USA; ^4^ Service of Statistics IRCCS Istituto Centro San Giovanni di Dio Fatebenefratelli Brescia Italy; ^5^ Department of Economics and Management University of Brescia Brescia Italy; ^6^ Department of Clinical Neurosciences Villa San Benedetto Menni Hospital, Hermanas Hospitalarias Como Italy; ^7^ Department of Biomedical Sciences Humanitas University Milan Italy; ^8^ Department of Mental Health AST PU Marche Fano Italy; ^9^ Department of Biotechnological and Applied Clinical Sciences (DISCAB) University of L'Aquila L'Aquila Italy

**Keywords:** borderline personality disorder, burden, caregivers, family connections, grief

## Abstract

Borderline personality disorder (BPD) has a strong impact not only on patients' lives but also on their families. The presence of an invalidating environment is one of the key factors in the etiology of BPD. This study evaluated the impact of the Family connections (FC) program on burden, grief, and other clinical variables in 202 caregivers and identified the profiles of participants who improved/deteriorated their levels of burden and grief. Findings from generalized linear mixed models showed significant reductions in burden, grief, depression, global psychological distress, and suppressed and expressed anger after FC intervention. Two classification trees were applied to test whether improvements in burden and grief were associated with age, gender and the improvements in other clinical variables. Caregivers reporting reduced depression were more likely to improve in both burden and grief. Moreover, younger participants showing increased depression had a 72.7% probability of being part of the improvement in burden and a 66.7% probability of being part of the improvement in grief. A decrease in depression and having a younger age were associated with positive gains for caregiving burden and grief. Longitudinally, ANOVAs showed positive changes in burden and grief as well as decreased depression, global psychological distress and suppressed anger were maintained at 4‐month follow‐up. Present findings improve our understanding of the utility of the FC program for caregivers of people with BPD. The impact of depressive symptoms' reduction and being younger on perceived burden and grief highlight the importance of exploring additional possible moderators of outcomes in FC intervention.

**Trial Registration:** NCT06076343; NCT06074289. Registered 10/10/2023

## 1 Introduction

Borderline personality disorder (BPD) is characterized by a pattern of instability in interpersonal relationships, self‐image, and affect, and is accompanied by impulsive behaviors (APA 2022). Individuals with BPD are more susceptible to engaging in self‐harming behaviors or attempting suicide. Due to its clinical features, BPD not only profoundly impacts the lives of those diagnosed, but also extends its influence to their families. According to the biosocial or transactional theory of BPD development (Fruzzetti, Shenk, and Hoffman 2005; Linehan 1993), the core feature of the disorder is emotion dysregulation, stemming from the interplay between biological emotional vulnerability and an invalidating environment.

While numerous patients recount experiencing diverse adverse events during childhood (Hengartner et al. 2013; Zanarini et al. 1997), the invalidating social and family environment may not be explicitly traumatic. However, it is marked by a pervasive challenge in comprehending, managing and validating emotions, feelings and behaviors of individuals with BPD.

Caregivers of people with BPD and related problems showed higher levels of somatic and psychological suffering compared to the general population (Scheirs and Bok 2007; Seigerman et al. 2020) and they reported the feeling of “living on tiptoes” due to individuals' with BPD unpredictability, sense of perpetual crisis and worries (Ekdahl et al. 2011). Moreover, the rates of objective and subjective burden among carers of individuals with BPD were higher than that experienced by significant others of people with other mental disorders (Bailey and Grenyer 2013; Kirtley et al. 2019; Seigerman et al. 2020). Several studies reported feelings of powerlessness (Bauer et al. 2012; Ekdahl et al. 2011), lifelong grief (Bailey and Grenyer 2014), interpersonal difficulties (Giffin 2008), and social stigma (Akbari et al. 2018; Bailey and Grenyer 2014; Hoffman et al. 2005; Kirtley et al. 2019) among carers of people with BPD. The family environment of people with a diagnosis of BPD showed high levels of expressed emotion such as criticism and emotional over‐involvement (Bailey and Grenyer 2015; Kirtley et al. 2019; Seigerman et al. 2020).

Other studies highlighted that family members reported the need to improve their knowledge about BPD (Kay et al. 2018), and have faced significant challenges when interacting with mental health services (Bauer et al. 2012; Lawn and McMahon 2015). The American Psychiatry Association guidelines for BPD treatment (APA 2001) and the most recent guidelines of the National Institute for Health and Clinical Excellence (NICE 2009) underlined the importance of an integrated approach in the treatment of BPD, indicating the intervention for families as a key element to support better clinical outcomes of their loved ones.

Family connections (FC) is a multi‐family education, skills training and support program developed by Alan Fruzzetti and Perry Hoffman, based on their extensive clinical research experience (Hoffman et al. 2005; Hoffman, Fruzzetti, and Buteau 2007). The FC program is rooted in the principles of Dialectical Behavior Therapy (DBT), which stands as one of the most empirically supported programs for relatives and caregivers of patients with BPD (Linehan 1993). Comprising 12 weekly sessions, the FC program is organized into six modules, each designed with specific objectives and practical exercises. These modules include an introduction, family psychoeducation, relationship mindfulness skills, family environment skills, validation skills, and problem management skills. Specifically, the FC program endeavors to educate caregivers about BPD in general, acquaint them with the biosocial theory of emotion vulnerability and dysregulation, and support them in understanding the behaviors of their family members with BPD and the dynamics of family functioning. The program further instills a set of skills derived from DBT, empowering caregivers to foster a mutually validating environment and enhance communication within the family. Additionally, the program strives to facilitate mutual social support among participants.

The initial study of FC, led by Hoffman et al. (2005), revealed positive changes in family members' burden, grief, and mastery post‐intervention. Positive changes in grief and mastery were sustained at a 3 month follow‐up, while burden continued to decrease. A subsequent replication study by the same authors (Hoffman, Fruzzetti, and Buteau 2007) not only confirmed the initial findings but also reported a reduction in depression scores post‐intervention. At a 3 month follow‐up, burden and depression remained stable, while grief scores continued to decrease. Following these studies, several other uncontrolled clinical trials of the FC program demonstrated its beneficial effects on levels of burden, grief, anxiety, and depression, as well as on participants' perceived sense of mastery, empowerment, well‐being, and family functioning.

In 2009, a pilot study by Rajalin and colleagues showed significant improvements in burden and well‐being among caregivers of individuals who had attempted suicide. In 2017, Flynn and colleagues compared a group (*n* = 51) who received a 12‐week FC program to an optimized treatment‐as‐usual group (*n* = 29), wherein participants received a 3‐week psychoeducational course about BPD. This comparison revealed positive changes for total scores on burden and grief that were maintained at 12–19 months after the intervention. In 2019, Liljedahl and colleagues in a non‐randomized comparison study found no significant differences between two types of FC programs for caregivers of individuals receiving DBT, namely 12‐week standard FC training (*n* = 34) and a two‐weekend intensive FC program (*n* = 48), in terms of improvements in burden, well‐being and overall family functioning. Nevertheless, both programs showed positive gains sustained at six to 7‐months post‐intervention. More recently, Boritz et al. (2021) showed statistically significant improvements over time in burden, grief, coping, and other secondary outcomes after an FC program among 94 caregivers of youth with diverse mental health challenges living in three different geographical locations in Canada. Although its findings have not yet been published (Fernández‐Felipe et al. 2020), a recent randomized controlled study has the potential to contribute to a more detailed understanding of the benefits of FC.

While FC stands out as one of the programs gathering substantial empirical support, certain authors have highlighted the limited evidence on peer‐delivered FC and the impact of the FC program among family members of individuals with a personality disorder and other comorbid conditions or those exhibiting subthreshold features of BPD. Additionally, there are concerns regarding the dissemination of FC to diverse cultural contexts and countries characterized by distinct mental health systems and resources.

This study marks the initial attempt to introduce and assess the application of the FC protocol in the Italian context. We initiated a pilot ecological study to gauge the acceptability of the FC program among participants, specifically caregivers of individuals with BPD and related issues, receiving treatment in various Italian mental health centers. Acknowledging the general constraints on funding that often limit the availability of group interventions within mental health services, it is crucial to underscore the significance of evaluating the acceptability of a brief intervention tailored to the specific characteristics of caregivers of individuals with BPD. This evaluation aims to facilitate a broader implementation of such interventions in similar settings across the country.

The main aim of this study was to evaluate the impact of a 12‐week FC program in real‐world practice on two main outcomes: perceived burden and grief among a sample of family members of patients with a diagnosis of BPD. Furthermore, we sought to evaluate the impact of FC program on other clinical variables (secondary outcomes): depression, alexithymia, global psychological distress, family functioning, and feelings of anger (expressed through verbal or physical behavior and suppressed anger). We also tested whether improvements in the two main outcomes (burden and grief scores) were associated with age, gender and the improvements in other clinical variables. Furthermore, we delved into the longitudinal impact of the FC program on a subset of family members who underwent the assessment at all three designated time points.

## 2 Method

### 2.1 Design and Sample

This prospective, single‐arm, open‐label study involved a sample of 233 relatives (caregivers) from 136 distinct families, each of which had a member diagnosed with BPD. Of these, 218 relatives completed the intervention, and 202 underwent a comprehensive pre‐post assessment. The participants were recruited from three different Italian mental health centers: IRCCS Centro San Giovanni di Dio Fatebenefratelli in Brescia, Fano Department of Mental Health in Fano, and the Mental Health Center “Benedetto Menni” in Albese con Cassano. Inclusion criteria were as follows: being 18 years old or older, being a caregiver or significant other of an individual with a diagnosis of BPD, and providing written consent. Potential participants were excluded from the study if they reported having an acute mental health condition interfering with group participation at that time. The socio‐demographic characteristics (age, sex, education) did not exhibit notable differences across centers. Additionally, the FC program was standardized across all centers, ensuring consistency in data collection. Therefore, the data collected from the three recruitment sites can be regarded as a single, homogeneous sample without compromising generalizability.

### 2.2 Ethics

The study was approved by the local ethics committee “Comitato Etico IRCCS Istituto Centro San Giovanni di Dio—Fatebenefratelli” in Brescia (reference number 98/2016, December 15, 2016 for Brescia; reference number 37/2018, May 9, 2018 for Fano, Leno and Albese con Cassano). All participants signed an informed consent form. The Leno site withdrew from the study due to changes in organizational needs before the commencement of the recruitment phase.

### 2.3 Evaluation Measures

Family members underwent an assessment including socio‐demographic information and a comprehensive battery of self‐report questionnaires. The battery included various measures as follows.

#### 2.3.1 Primary Intervention Outcomes

##### 2.3.1.1 Burden Assessment Scale (BAS)

The BAS scale was used to measure the construct of the burden of providing ongoing care to individuals with mental health problems (Reinhard et al. 1994). In this study, the total BAS score was used, with higher scores indicating higher levels of perceived burden, and exhibiting good reliability (Cronbach's alpha coefficient = 0.89).

##### 2.3.1.2 Grief Scale (GS)

GS is a 15‐item questionnaire that assesses the experience of grief associated with having a loved one with mental illness (Struening et al. 1995). Higher scores represent more intense experiences of grief. In this study, the statistical analysis revealed that the GS scale demonstrated satisfactory internal consistency, with Cronbach's alpha = 0.92.

It's worth noting that neither the BAS scale nor the GS scale have been formally validated in Italian. However, for both scales, a back translation was conducted by a professional translator, to ensure accuracy and consistency in the Italian version used in the study.

#### 2.3.2 Secondary Intervention Outcomes

##### 2.3.2.1 Family Functioning Questionnaire (FF Questionnaire)

The FF is a 24‐item questionnaire that assesses family functioning (Roncone et al. 2007). For the purpose of this study, we used the total FF score, with higher scores indicating a greater occurrence of positive family‐related behaviors. In this study, the questionnaire showed good psychometric properties, with a Cronbach's alpha coefficient = 0.83 for the total score.

##### 2.3.2.2 Symptom Check List‐90 (SCL‐90)

The SCL‐90 scale was used to assess psychological problems and psychopathological symptoms (Derogatis, Lipman, and Covi 1977; Prunas et al. 2012; Rief and Fichter 1992). For the purpose of this study, we used the Global Severity Index (GSI), which is the mean value of all of the items, and is considered a measure of global psychological distress (in this sample, Cronbach's alpha coefficients = 0.97).

##### 2.3.2.3 Beck Depression Inventory‐II (BDI‐II)

The BDI‐II questionnaire was used to assess the severity of depressive symptoms (Beck, Steer, and Brown 1996; Ghisi et al. 2006). BDI‐II is a 21‐item self‐report questionnaire with higher scores reflecting higher levels of depression. In our study, the scale showed good internal consistency, as evidenced by Cronbach's alpha coefficients = 0.89.

##### 2.3.2.4 Toronto Alexithymia Scale 20 (TAS‐20)

The TAS‐20 scale was used to assess alexithymia (Bagby, Parker, and Taylor 1994; Bressi et al. 1996). It is a 20‐item scale with higher scores indicating higher levels of alexithymia. The scale showed good internal consistency (Cronbach's alpha coefficients = 0.83).

##### 2.3.2.5 State‐Trait Anger Expression Inventory (STAXI‐2)

The STAXI‐2 scale is a 57‐item inventory measuring the experience, expression, and control of anger (Comunian 2004; Spielberger 1999). For the purpose of this study, the Anger Expression‐Out (STAXI‐2 ER/OUT) subscale was used to measure the frequency in the expression of feelings of anger verbally or physically, along with the Anger Expression‐In (STAXI‐2 ER/IN) that measures how often the individual experiences anger, suppresses or withholds it, instead of expressing it. The Cronbach's alpha for STAXI‐2 ER/OUT and for STAXI‐2 ER/IN was 0.71 and 0.69, respectively.

### 2.4 Procedure

Participants were family members of outpatients or inpatients with a diagnosis of BPD who were actively utilizing the mental health services of the three study sites or other local mental health centers at the time of recruitment. Informed written consent for participation in the research study was obtained by the researchers at the outset of the investigation. The assessment process involved three distinct time points. At the initial stage (T0), participants were tasked with completing the outcome measures at home and they were kindly requested to return the completed questionnaires to the research team before the second session of the program. Following the conclusion of the FC program, at the post‐intervention timepoint (T1), participants were asked to replicate the same set of measures on the day immediately following the final session of the intervention. Four months after the completion of the FC program, participants were contacted via telephone as a gentle reminder to complete the follow‐up questionnaires (T2). These follow‐up questionnaires were conveniently to be returned via email within a 1‐week timeframe. In the event that the completed measures were not returned within this period, participants received another reminder call to encourage the completion and timely submission of the questionnaires.

### 2.5 Treatment Program

FC is a 12‐week manualized educational, skill and support program delivered in group settings to family members of individuals with BPD (90 min per week). The program includes six modules: (1) Orientation and psycho‐education on the most recent available information and research on BPD; (2) Psycho‐education on BPD's course of development and available treatments, comorbidity, and emotion dysregulation and transactional model for BPD and emotion dysregulation; (3) Individual skills and relationship skills to promote participant emotional well‐being (emotional self‐management, mindfulness, relationship mindfulness, letting go of judgments and decreasing emotional vulnerability); (4) Family and relationship skills to improve the quality of family relationships and interactions (letting go of blame and anger, and acceptance skills); (5) Validation (accurate and effective self‐expression, validation strategies); (6) Problem management skills (e.g., defining problems effectively, collaborative problem solving, balance between acceptance and change; Fruzzetti and Hoffman 2004). The FC intervention program was led by two cognitive‐behavioral therapists (leader and co‐leader) who received training in the FC program and DBT.

FC is provided on a voluntary basis, in collaboration with the National Education Alliance for BPD association (NEA‐BPD).

### 2.6 Statistical Analysis

Descriptive statistics are presented in terms of means and standard deviations (SDs) for continuous variables and in terms of frequency and percentage (%) for categorical variables. Generalized linear mixed models (GLMMs) were applied to assess changes from baseline to post‐intervention for the clinical scales by including intercept as a random effect in order to take into account the baseline variability of the participants. An additional random effect was included (family effect) for managing the clustering nature of some caregivers: some BPD individuals came from the same family and shared the same caregivers. Due to literature evidence and current data findings, all models were adjusted for age and gender. Change (baseline T0‐ post‐intervention T1) in the two main outcomes (BAS and GS) and in the other clinical scales was dichotomized into two categories: *improved* vs. *deteriorated*, where improved was defined by a positive (T0–T1) change and deteriorated by a negative (T0–T1) change. The clinical scales displaying significant alterations between T0 and T1, along with socio‐demographic traits, were examined as factors linked to feelings of burden and grief. In order to identify the profiles of caregivers who improved/deteriorated in their level of burden and grief in relation to the improved/deteriorated in the other clinical variables, two Classification Trees (CT; one for BAS and one for GS) were applied. The classification trees are a popular machine learning technique, particularly useful for classification problems and profile identification. In detail, the algorithm divides the predictor space using splitting rules, resulting in a tree‐like structure with all observations contained in the top node. Subjects are subsequently divided into subgroups until a predefined stopping criterion is met, leading to the formation of final nodes, or leaves. These nodes provide information about the most probable subject category and are connected by branches established by the splitting rules. The CT is explored from the top node to the leaves, presenting subject percentages and the likelihood of belonging to a response category (Hastie et al. 2009). The critical response‐linked variables function as discriminants, classifying subjects based on their categories or thresholds and associating them with either improved or deteriorated outcomes. The CT was carried out by the classification and regression tree (CART) growing method and a pruning procedure based on maximum risk difference was applied to avoid overfitting (https://www.ibm.com/docs/en/spss‐statistics/26.0.0?topic=trees‐creating‐decision). The CT was trained on a subset (60% of the whole sample) and the accuracy was evaluated on a test set (40% of the whole sample). Longitudinal analysis of clinical scales on a subsample of caregivers having completed the evaluation at all the three‐time points was analyzed using ANOVAs for repeated measures with Bonferroni post hoc p‐value adjustments. Univariate logistic models were performed to assess the association between burden change (dichotomized—improved vs. deteriorated—BAS change variable as dependent variable) and the socio‐demographic and clinical scales (dichotomized as improved/deteriorated). A multiple logistic regression model was then applied, including all the variables that were significant in the univariate logistic models. All statistical analyses were performed with SPSS statistics software, version 27. Significance was set at *p* < 0.05.

## 3 Results

### 3.1 Socio‐Demographic and Clinical Characteristics

The current study initially included a sample of 233 relatives of people with BPD (142 males, age *M* = 53.3, *SD* = 10.9). Assessments were planned at three‐time points: 87% of participants (*n* = 202) returned the post‐program questionnaires (i.e., 87% completed the baseline and post‐program evaluation). The dropout rate was about 6.5% (*n* = 15). Among those who completed the intervention, 16 participants did not return the pre‐post evaluation questionnaires. Moreover, 53% (*n* = 123) completed all three evaluations (i.e., returning the 4‐month follow‐up questionnaires). Considering the main aim of the study was to evaluate the impact of a 12‐week FC program in real‐world practice, we focused the analyses on the subsample who completed both the baseline (T0) and post‐program (T1) evaluations. The subsample of 202 relatives who completed the intervention and with complete evaluations at both T0 and T1 consisted of 79 males (39.1%) and 123 females (60.9%), with a mean age of 53.2 (SD = 10.9; see Table 1). Descriptive statistics on the whole sample were reported in Table S1.

**TABLE 1:** Socio‐demographic and clinical characteristics of the sample at baseline, *N* = 202.

	*n*	%	M	SD	M	SD
Gender						
Males	79	39.1				
Females	123	60.9				
Relationship type						
Partner	20	9.9				
Parent	150	74.2				
Brother/Sister	14	6.9				
Son/Daughter	10	5.0				
Other	8	4.0				
Marital status						
Single	15	8.1				
Married	116	62.4				
Divorced	38	20.4				
Widow	9	4.8				
Cohabitant	8	4.3				
Occupational status						
Unemployed	24	12.9				
Retired/Disabled	23	12.4				
Employed	131	70.4				
Student	3	1.6				
Housewife	5	2.7				
Age (years)			53.2	10.9		
Education (years)			12.8	3.9		
BDI‐II			14.1	9.1	0.7	0.4
TAS			53.6	13.7	2.7	0.7
SCL‐90			70	47.4	0.8	0.5
STAXI‐2 ER/IN			18.5	4	2.3	0.5
STAXI‐2 ER/OUT			15.4	3.5	1.9	0.4
BAS			45.9	11.1	2.3	0.6
GS			53.2	12.8	3.5	0.9
FF			66.2	8.8	2.8	0.4

*Note:* In the third and fourth column are reported the means and standard deviations on the same range of the Likert scale: BDI‐II [0–3]; TAS [1–5]; SCL‐90 [0–4]; STAXI‐2 ER/IN [1–4]; STAXI‐2 ER/OUT [1–4]; BAS [1–4]; GS [1–5]; FF [1–4].

Abbreviations: BAS, Burden Assessment Scale; BDI‐II, Beck Depression Inventory‐II; FF, Family Functioning Questionnaire; GS, Grief Scale; SCL‐90, Symptom Check List‐90; STAXI‐2 ER/IN, State‐Trait Anger IN Expression Inventory; STAXI‐2 ER/OUT, State‐Trait Anger OUT Expression Inventory; STAXI‐2 ER/OUT, State‐Trait Anger OUT Expression Inventory; TAS, Toronto Alexithymia Scale.

### 3.2 Effect of FC Program on Clinical Variables

GLMMs (all adjusted for age and gender) were performed to evaluate changes from T0 to T1 of the eight assessment scales. Results showed that both the main outcome measures, BAS and GS, significantly decreased (i.e., showed significant improvement) from T0 to T1 (time coefficient = −0.29 for BAS and − 0.35 for GS, *p* < 0.001 for both scales). Significant improvements were found also for depression (BDI‐II scores, *p* < 0.001), SCL‐90 (*p* = 0.008), STAXI‐2 ER/IN (*p* = 0.024) and STAXI‐2 ER/OUT scores (*p* = 0.012). No significant changes were found for the FF questionnaire and TAS scale (Table 2).

**TABLE 2:** Generalized linear mixed models output for the longitudinal assessment from baseline to post intervention.

Models [dependent and independent – fixed‐variables]	Beta coefficient^a^, ^b^	*p*
Model dependent variable: BAS		
Time	−0.287	**< 0.001**
Age	+0.001	0.820
Gender	−0.132	**0.015**
Model dependent variable: GS		
Time	−0.354	**< 0.001**
Age	+0.013	**0.001**
Gender	−0.269	**0.002**
Model dependent variable: FF		
Time	+0.035	0.330
Age	+0.001	0.572
Gender	+0.023	0.536
Model dependent variable: BDI‐II		
Time	−0.171	**< 0.001**
Age	+0.002	0.342
Gender	−0.154	**< 0.001**
Model dependent variable: TAS		
Time	−0.063	0.301
Age	+0.001	0.660
Gender	+0.086	0.168
Model dependent variable: SCL‐90		
Time	−0.125	**0.008**
Age	+0.001	0.934
Gender	−0.140	**0.004**
Model dependent variable: STAXI‐2 ER/IN		
Time	−0.118	**0.024**
Age	+0.001	0.889
Gender	+0.050	0.290
Model dependent variable: STAXI‐2 ER/OUT		
Time	−0.106	**0.012**
Age	−0.002	0.292
Gender	−0.013	0.772

*Note:* Bold values represent statistically significant results that is  ≤ 0.05.

Abbreviations: BAS, Burden Assessment Scale; BDI‐II, Burden Assessment Scale; FF, Family Functioning Questionnaire; GS, Grief Scale; SCL‐90, Symptom Check List‐90; STAXI‐2 ER/IN, State‐Trait Anger Expression Inventory‐2 Expression/In; STAXI‐2 ER/OUT, State‐Trait Anger Expression Inventory‐2 Expression/Out; TAS, Toronto Alexithymia Scale.

^a^ Baseline as reference time.

^b^ Female as reference category. In models with significant gender and Time, a further investigation of the interaction effect Gender × Time was carried out. None of these interaction effects were significant.

Follow‐up evaluation: improvements in burden and grief were maintained at the 4‐month follow‐up. Similar results were found for the BDI‐II, SCL‐90 and STAXI‐2 ER/IN. Conversely, STAXI‐2 ER/OUT worsened at follow‐up (moving toward scores close to pre‐intervention). In addition, no improvements were found for the FF questionnaire and TAS total mean scores at T1, albeit small positive changes were found at T2 (see Table S2).

### 3.3 Relatives' Profiles in Terms of Burden and Grief Changes

**FIGURE 1:**
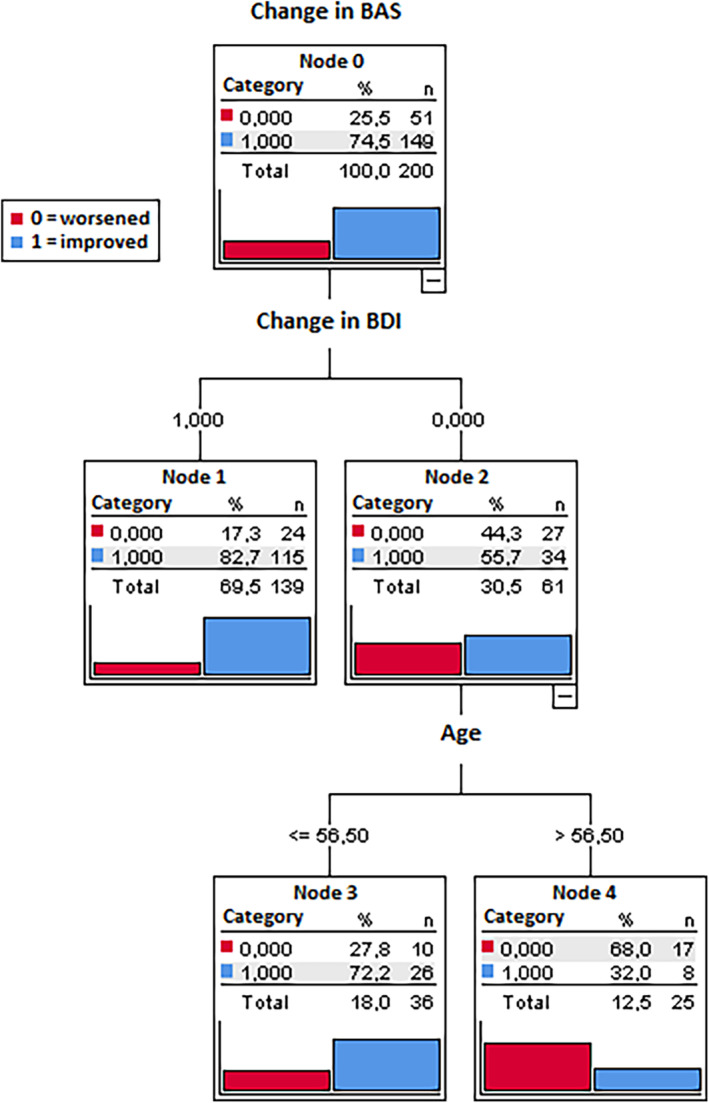
Classification tree for burden (BAS scale) change. Results of CT applied on BAS (post‐pre intervention) change (worsened vs. improved). Total percentage of accuracy = 79% (accuracy for improved: 94.6%, accuracy for worsened 33.3%); Risk estimate 0.21 (standard error risk estimate = 0.029).

**FIGURE 2:**
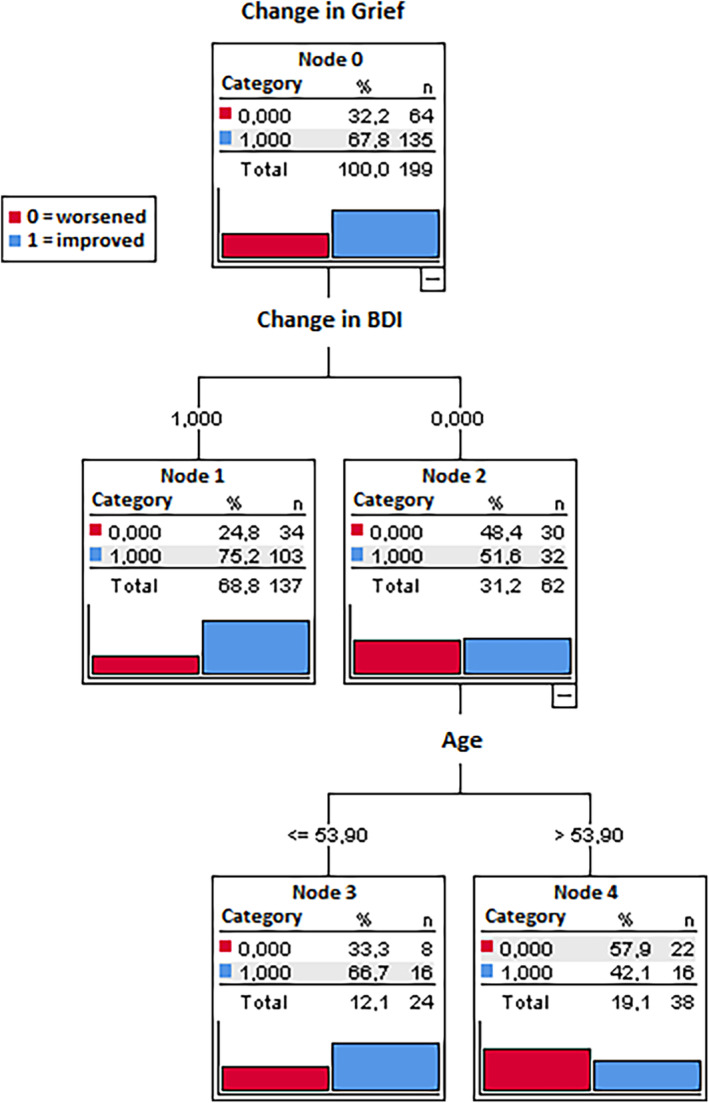
Classification tree for grief (GS scale) change. Results of CT applied on GS (post‐pre intervention) change (worsened vs. improved). Total percentage of accuracy = 70.9% (accuracy for improved: 88.1%, accuracy for worsened 34.4%); risk estimate 0.29 (standard error risk estimate = 0.032).

To identify the profiles of relatives who improved versus deteriorated on their levels of burden (BAS) and grief (GS), in relation to the improved/deteriorated levels on the other clinical variables, two classification Trees—CT—(one for BAS and one for GS) were applied, as described above. CT analysis revealed that depression (BDI‐II) and age exhibited the strongest associations with both BAS and GS. Subjects who improved in BDI‐II scores (69.5% of the total participants) had an 82.7% probability of also experiencing improvement on the BAS, and only a 17.3% probability of deterioration. Among individuals who deteriorated on the BDI‐II (30.5% of the total participants), subjects older than 56.5 years of age (12.5%) were more likely to deteriorate on their BAS scores (68% probability, vs. probability of only 32% to improve); alternatively, among who deteriorated on the BDI‐II, subjects younger than 56.5 years of age (18%) were more likely to improve on their BAS scores (72.7% probability, vs. probability of only 27.8% to deteriorate; Figure 1). In other words, individuals with increased scores in BDI‐II and aged over 56.5 years are less likely to be part of the group of participants that improved post‐intervention on burden. Similarly, subjects who improved on the BDI‐II had a 75.2% probability to improve on their GS scores, and only a 24.8% probability of deteriorating. Among individuals who got worse on the BDI‐II, subjects over 53.9 years of age had a 57.9% probability of deteriorating on their GS scores (vs. a probability of only 42.1% to deteriorate). Conversely, individuals who got worse in the BDI‐II but who were less than 53.9 years old had a 66.7% probability to improve on their GS scores (vs. a probability of only 33.3% to deteriorate; Figure 2). In other words, individuals with elevated scores in BDI‐II and aged over 53.9 years are less likely to be part of the group of participants who showed improvement in grief post‐intervention.

As indicated by further logistic regression analyses (univariate and multiple models) the CT findings were confirmed. Indeed, results showed that age and the two dichotomous (improved vs. deteriorated) BDI‐II and SCL‐90 variables were the most related to burden change (Table S3). Also, change in grief was directly and positively related to burden change. In the multiple models, age (OR = 0.97, 95% CI: 0.92–0.99), BDI‐II (OR = 3.07, 95% CI: 1.70–7.72) and GS (OR = 2.34, 95% CI: 1.13–4.83) remained as the most prominent variables associated with burden, while SCL‐90 was no longer associated, mainly due to its correlation with BDI‐II (see Table S5). In detail, an increase of 1 year of age was associated with a decreased likelihood of being in the group that had a reduced burden. In other words, each additional increase of 1 year in age was associated with a 3% decrease in the odds of improving on the BAS scale, while being in the group of subjects with improved BDI‐II or improved GS increased the odds (3.1 times and 2.3 respectively) of improving on the BAS. Similar results were found for the dichotomized (improved vs. deteriorated) grief variable (Table S4).

## 4 Discussion

The present study investigated the impact of a 12‐week FC program for family members of individuals with BPD in three different sites located in the northern and central regions of Italy. In line with previous studies (Flynn et al. 2017; Hoffman et al. 2005; Hoffman, Fruzzetti, and Buteau 2007; Liljedahl et al. 2019), the FC program showed statistically significant reductions from pre‐ to post‐intervention in overall burden, grief, depression, global psychological distress and anger, accounting for gender and age. This is in line with other studies showing that depression decreased from pre‐ to post‐intervention (Flynn et al. 2017; Hoffman, Fruzzetti, and Buteau 2007; Rajalin et al. 2009), as well as global psychological distress (Liljedahl et al. 2019) and anger (Nguyen 2020).

Contrary to previous studies (Liljedahl et al. 2019; Sheikhan et al. 2021), no improvements in global family functioning post‐intervention were found, although a significant gain was observed at follow‐up. One possible reason for this inconsistency may be that family functioning requires a longer period of skills practice to see considerable changes. Other FC studies have demonstrated similar improvements from post‐intervention to a follow‐up 6 months later (cf. Hoffman et al. 2005; Hoffman, Fruzzetti, and Buteau 2007). Although it was evaluated only on a sub‐sample of 123 family members, it is important to note that statistically significant improvements in BAS, GS, TAS and BDI‐II scores were sustained at 4 months after completion of the program, providing encouraging findings in terms of FC medium‐term outcomes. Considering anger subscales, we found that expressed anger and suppressed anger showed different tendencies. STAXI‐2 ER/IN improved during the follow‐up period. In contrast, STAXI‐2 ER/OUT decreased between baseline and the end of treatment, and then at follow‐up, moving back toward pre‐intervention levels. Individuals with a higher frequency of experiencing feelings of anger expressed verbally or physically could benefit significantly from the weekly sessions and perhaps the practice of exercises between sessions. These individuals could need more support and practice to generalize their improvements, and thus be more negatively affected by the group ending, and the reduced practice and support network that follows after the program ends. It is plausible that individuals with higher expressed anger were able to learn some strategies to regulate anger emotions, but that the FC program's duration was not enough to stabilize their skill use after the end of the intervention. In general, as pointed out by other authors (Hoffman, Fruzzetti, and Buteau 2007; Liljedahl et al. 2019), additional FC sessions or booster sessions for family members who completed the FC program could be beneficial in order to support their changes over time. Further investigations are needed to evaluate the enduring effects of the FC program on various facets of anger among caregivers of individuals with BPD.

The second aim of this study was to identify the variables most strongly linked to improvements in perceived burden and feelings of grief associated with the complex challenges of caring for a loved one with significant and chronic BPD‐related problems. Firstly, we observed that there is a relationship between changes in depression and burden and grief scores. Indeed, individuals who reported reduced depression after the intervention were more likely to show improvements in both burden and grief scores. Secondly, individuals who reported higher depression, but were younger than 56.5 years of age, had a 72.2% probability of being part of the “improved in BAS” group. Lastly, individuals who reported higher depression and were younger than 53.9 years of age had a 66.7% probability of being part of the “improved in GS” group. These findings showed that depression has varying degrees of association with perceived burden and grief across different age cohorts. Specifically, 115 individuals who showed improvements in BAS and 103 individuals who improved in GS achieved better outcomes in terms of depression. This suggests that interventions capable of addressing depression may have a positive impact on reducing perceived burden and grief among carers of individuals with BPD. On the other hand, despite reporting higher depression scores, a low percentage of carers had positive outcomes in burden and grief post‐intervention (34 for BAS and 32 for GS). Similarly, a cross‐sectional study (Hoffman et al. 2003), found that younger family members reported higher burden and depression than older ones. In another study (Scheirs and Bok 2007) older carers reported higher depression scores. However, a recent study (Kirtley et al. 2019) did not find any significant relationships between age and burden variance. In our study, we found that among carers who reported higher depression scores post‐ than at pre‐intervention, 26 out of 34 individuals younger than 56.5 years old improved in BAS scores, and 16 out of 32 individuals younger than 53.9 years old improved in GS scores, suggesting that FC program may be effective among individuals below these ages in reducing burden and, to a lesser extent, feelings of grief, regardless their level of depression. Interestingly, a recent cross‐sectional study among 287 caregivers of people with a personality disorder (Bailey and Grenyer 2014) found that greater scores in burden and grief were associated with a younger age of carers.

In our study, we hypothesize that younger carers, although they experience similar amounts of burden and grief compared to older ones, are less impaired in part because they have had a shorter length of caregiving and, presumably, of psychological distress. It is possible that older caregivers had had a greater duration of caregiving and, presumably, that is associated with higher psychological distress (i.e., longer periods of burden, more losses). Thus, age may be confounded with a duration of caregiving, and further research is needed to discern these relationships. These findings demonstrate the importance of providing specific interventions to support older caregiving relatives of people with BPD when they suffer from higher levels of depression.

### 4.1 Strengths and Limitations

The present study includes both strengths and limitations. The main strength of this study is the relatively large sample size, with a medium‐term follow‐up. Furthermore, we assessed several clinical aspects and their relationships, including a broad range of participants with few exclusions. Relatives with BPD were involved in a variety of treatments (including no treatment), increasing the study's ecological validity. Also, to reduce selection bias, participants were consecutively selected. However, a number of limitations of the current study should be considered. First, this was a study conducted in real‐world practice setting and it was not possible to recruit a control group or waiting‐list group, limiting the generalizability of the data to some extent. In addition, excluding participants who reported experiencing acute mental health conditions at the moment of enrollment could also limit the generalizability of findings, as the sample may not fully represent individuals with such conditions, preventing them from evaluating clinically significant changes. Moreover, the rate of collected follow‐up was only 53%. Further studies should include the use of digital data collection systems to increase questionnaires' completion rates and administration accuracy. Thus, longer‐term results should be considered with caution. Demographic information on individuals with BPD, such as age and gender, along with their clinical characteristics (e.g., severity and duration of illness, number of hospitalizations, type of treatment they were receiving, suicidal attempts, presence of self‐harming behaviors, etc.) was not collected in all the study sites. Further studies should evaluate the impact of these relevant covariates on caregivers' burden and grief. In this study, in contrast to many FC studies, FC was not peer‐led. Rather, FC was led by trained clinicians. However, our findings show similar outcomes to previous studies that used clinicians as group leaders, as well as those that utilized professionals to lead FC groups. Moreover, a further multi‐centered study should include family members in different regions across Italy to test the generalizability of our findings in various healthcare settings and locations. Finally, Italy is a country mainly grounded on a collectivistic and traditional culture that may influence the expression of BPD symptomatology and the process of treatment. For these reasons, studies on the impact of cultural aspects on the efficacy of the FC program are warranted.

## 5 Conclusions and Implications

Results of the present study highlight the importance of including families in the treatment of BPD for many reasons, but further research is needed to assess the indirect (transactional) impact of FC with caregivers on BPD patients and on the relationships between caregivers and BPD patients. The biosocial theory or transactional model includes an invalidating social and family environment among the contributing factors for the development and maintenance of BPD. The present study provides further evidence that including family members in the treatment process should be essential. However, there may be additional participant factors (moderators) that facilitate or attenuate outcomes. Present findings with respect to age highlight the importance of exploring additional possible moderators of outcomes. In addition, further studies including different formats of FC are needed, such as evaluating whether different types of group leaders affect outcomes (e.g., peer facilitators who are FC graduates, service providers, or a combination of professional and non‐professional staff). Ultimately, further experimental evidence studies for supporting a conclusion of causation between depression and perceived burden and grief will be useful to provide comprehensive evidence of the utility of the FC program.

The present study significantly enriches the existing literature by shedding light on the associations between the FC program and the well‐being of relatives of individuals with BPD. The overall association of FC with a range of outcomes was significant, as hypothesized.

In conclusion, FC demonstrated a strong association with the reduction of caregiving burden, grief, depression, global psychological distress, and anger among participants in three distinct sites within Italian mental health services. A reduction in depressive symptoms and a younger age emerge as significantly associated factors with more favorable perceptions of burden and grief following an FC intervention among caregivers of individuals with BPD. These findings highlight the relevance of taking these factors into account in the realm of caregiving support.

## Conflicts of Interest

The authors declare no conflicts of interest.

## Supporting information


Table S1.–S5.

## Data Availability

A datasheet reporting the clinical variables included in the manuscript will be available on request on ZENODO repository (DOI: 10.5281/zenodo.7261504). In compliance with our institute's privacy protocols, any information that could threaten participants' anonymity has been deleted.
