# Supplementary material for: Family Connections: The Impact of an Education Program for Carers of Individuals With Borderline Personality Disorder in Italian Mental Health Services
Source: Fam Process. 2025 Jan 28;64(1):e13098. doi: 10.1111/famp.13098 (PMC11774136; doi:10.1111/famp.13098)
Supplement: Supplementary file 1 — Table S1.–S5. [file FAMP-64-0-s001.docx]

**SUPPLEMENTARY MATERIALS**

|  | **Table S1** | | | | | | | | | | | | | | |  |
| --- | --- | --- | --- | --- | --- | --- | --- | --- | --- | --- | --- | --- | --- | --- | --- | --- |
|  | *Socio-demographic and clinical characteristics of the whole sample at baseline* | | | | | | | | | | | | | | |  |
|  |  | | | | | | | | | | | | | | |  |
|  | | | |  | | | | Socio-demographic feature by center | | | | | | | |  |
|  | |  | | N=233 | | | | 1 | | | 2 | | 3 | | |  |
|  | |  | | n | | % | | n | | % | n | % | n | % | |  |
| Gender | |  | |  | |  | |  | |  |  |  |  |  | |  |
| Males | |  | | 91 | | 39.1 | | 49 | | 53.8 | 8 | 8.8 | 34 | 37.4 | |  |
| Females | |  | | 142 | | 60.9 | | 84 | | 59.2 | 10 | 7 | 48 | 33.8 # | |  |
|  | |  | |  | |  | |  | | |  | |  | | |  |
|  | |  | | M | | SD | | M | | SD | M | SD | M | | SD |  |
|  |  |  |  |  | |  |  |  |  |  |  |  |  |  |  |  |
| Age | |  | | 53.3 | | 10.9 | | 53.9 | | 12.4 | 52.1 | 13.4 | 52.4 | | 13.5 # |  |
| Education years | |  | | 12.7 | | 3.8 | | 12.7 | | 3.8 | / | / | 12.7 | | 3.8 # |  |
|  | |  | |  | |  | |  | | |  | |  | | |  |
|  | |  | | n | | % | |  | | | | | | | |  |
| Relationship with the patient | |  | |  | |  | |  | | | | | | | |  |
| Partner | |  | | 25 | | 10.8 | |  | | | | | | | |  |
| Parent | |  | | 171 | | 73.7 | |  | | | | | | | |  |
| Brother/Sister | |  | | 16 | | 6.9 | |  | | | | | | | |  |
| Son/Daughter | |  | | 10 | | 4.3 | |  | | | | | | | |  |
| Other | |  | | 10 | | 4.3 | |  | | | | | | | |  |
| Marital status | |  | |  | |  | |  | | | | | | | |  |
| Single | |  | | 17 | | 8.1 | |  | | | | | | | |  |
| Married | |  | | 132 | | 62.6 | |  | | | | | | | |  |
| Divorced | |  | | 42 | | 19.9 | |  | | | | | | | |  |
| Widow | |  | | 10 | | 4.7 | |  | | | | | | | |  |
| Cohabitant | |  | | 10 | | 4.7 | |  | | | | | | | |  |
| Occupational status | |  | |  | |  | |  | | | | | | | |  |
| Unemployed | |  | | 24 | | 11.5 | |  | | | | | | | |  |
| Retired/Disabled | |  | | 27 | | 13.0 | |  | | | | | | | |  |
| Employed | |  | | 146 | | 70.2 | |  | | | | | | | |  |
| Student | |  | | 4 | | 1.9 | |  | | | | | | | |  |
| Housewife | |  | | 5 | | 2.4 | |  | | | | | | | |  |
|  | |  | |  | |  | |  | | | | | | | |  |
|  | | M | SD | | M | | SD | |  | | | | | | | |
|  |  |  |  |  |  | |  |  |  |  |  |  |  |  |  |  |
| BDI-II | | 13.8 | 8.8 | | 0.7 | | 0.4 | |  | | | | | | | |
| TAS | | 52.9 | 13.7 | | 2.6 | | 0.7 | |  | | | | | | | |
| SCL-90 | | 68.3 | 46.6 | | 0.8 | | 0.5 | |  | | | | | | | |
| STAXI-2 ER/IN | | 18.4 | 4.2 | | 2.3 | | 0.5 | |  | | | | | | | |
| STAXI-2 ER/OUT | | 15.5 | 3.6 | | 1.9 | | 0.4 | |  | | | | | | | |
| BAS | | 45.8 | 11.1 | | 2.3 | | 0.6 | |  | | | | | | | |
| GS | | 52.9 | 13 | | 3.5 | | 0.9 | |  | | | | | | | |
| FF | | 66.4 | 9 | | 2.8 | | 0.4 | |  | | | | | | | |
|  | |  | |  | |  | |  | | | | | | | |  |
|  | *Note.* BDI-II = Beck Depression Inventory II; TAS = Toronto Alexithymia Scale; SCL-90 = Symptom Check List-90; STAXI-2 ER/IN = State Trait Anger IN Expression Inventory; STAXI-2 ER/OUT = State Trait Anger OUT Expression Inventory; STAXI-2 ER/OUT = State Trait Anger OUT Expression Inventory; BAS = Burden Assessment Scale; GS = Grief Scale; FF = Family Functioning Questionnaire. In the third and fourth column are reported the means and standard deviations on the same range of the Likert scale: BDI-II [0-3]; TAS [1-5]; SCL-90 [0-4]; STAXI-2 ER/IN [1-4]; STAXI-2 ER/OUT [1-4]; BAS [1-4]; GS [1-5]; FF [1-4].  # no statistical differences among center. | | | | | | | | | | | | | | |  |

| **Table S2** | | | | | | | | |
| --- | --- | --- | --- | --- | --- | --- | --- | --- |
| *Repeated measure Anova tests and post-hoc evaluation* | | | | | | | | |
|  | T0  n = 233 | | T1  n = 202 | | T2  n = 123 | |  |  |
|  | M | SD | M | SD | M | SD | p-value £ | Post-hoc £ |
| BAS | 2.3 | 0.6 | 2.0 | 0.6 | 2.0 | 0.6 | **<.001** | T0 vs T1 **<.001**  T0 vs T2 **<.001** |
| GS | 3.5 | 0.9 | 3.2 | 0.9 | 3.1 | 1.0 | **<.001** | T0 vs T1 **<.001**  T0 vs T2 **<.001** |
| FF | 2.8 | 0.4 | 2.8 | 0.4 | 2.9 | 0.4 | **.016** | T0 vs T2 **.017** |
| BDI-II | 0.7 | 0.4 | 0.5 | 0.4 | 0.5 | 0.5 | **<.001** | T0 vs T1 **<.001**  T0 vs T2 **<.001** |
| TAS | 2.85 | 0.7 | 2.75 | 0.6 | 2.72 | 0.6 | **.010** | T0 vs T2 **.014** |
| SCL-90 | 0.8 | 0.6 | 0.7 | 0.5 | 0.7 | 0.6 | **<.001** | T0 vs T1 **<.001**  T0 vs T2 **.006** |
| STAXI-2 ER/IN | 2.3 | 0.5 | 2.2 | 0.6 | 2.2 | 0.5 | **.006** | T0 vs T1 **<.022**  T0 vs T2 **.020** |
| STAXI-2 ER/OUT | 2.0 | 0.5 | 1.8 | 0.4 | 1.9 | 0.5 | **.005** | T0 vs T1 **.009** |
| *Note.* BAS= Burden Assessment Scale; GS= Grief Scale; FF= Family Functioning Questionnaire; BDI-II= Burden Assessment Scale; TAS= Toronto Alexithymia Scale; SCL-90= Symptom Check List-90; STAXI-2 ER/IN= State Trait Anger Expression Inventory-2 Expression/In; STAXI-2 ER/OUT= State Trait Anger Expression Inventory-2 Expression/Out. T0= baseline assessment; T1= post-intervention assessment; T2= 4 months follow-up assessment. £ ANOVA p-values and post-hoc evaluations for the n=123 participants having assessment in all three time points. | | | | | | | | |

| **Table S3** | | | | | |  |
| --- | --- | --- | --- | --- | --- | --- |
|  | | | | | |  |
| *Logistic regression models on perceived burden change (dichotomized BAS change variable as dependent variable)* | | | | | |  |
|  | Independent variables | Nagelkerke’s  R^2^ | P value | Odds ratio  OR | OR Confidence interval | |
| Univariate  models | Age | 0.036 | **.034** | **0.965** | **0.933 – 0.997** | |
|  | Sex | 0.019 | .109 | 0.591 | 0.311 – 1.124 | |
|  | Relationship with the patient | 0.058 | .134 | A – 14.167  B – 7.568  C – 9.167  D – 5.833 | **1.827 – 109**  **1.408 – 40**  **1.147 – 73**  0.696 – 48 | |
|  | Education (years) | 0.001 | .724 | 1.015 | 0.932 – 1.106 | |
|  | Diff_BDI-II | 0.108 | **<.001** | **3.535** | **1.788 – 6.989** | |
|  | Diff_SCL-90 | 0.041 | **.030** | **2.061** | **1.072 – 3.962** | |
|  | Diff_STAXI-2 ER/IN | 0.010 | .247 | 1.466 | 0.767 **–** 2.799 | |
|  | Diff_STAXI-2 ER/OUT  Diff_GS | 0.006  1.143 | .334  **<.001** | 0.725  **3.14** | 0.377 – 1.393  **1.611 -6.105** | |
| Multiple model | Age  Diff_BDI-II  Diff_SCL-90  Diff_GS | 0.182 | **.043**  **.005**  .698  **.022** | **0.968**  **3.067**  1.165  **2.336** | **0.923 – 0.992**  **1.699 – 7.715**  0.563 – 2.551  **1.130 – 4.833** | |
| *Note*. Diff_BDI-II= Change baseline-post intervention (T0-T1) in BDI-II (0= worsened, 1= improved); Diff_SCL90= Change T0-T1 in SCL-90 (0= worsened, 1= improved); Diff_STAXI-2 ER/OUT= Change T0-T1 in STAXI ER OUT (0= worsened, 1= improved); Diff_GS: Change T0-T1 in GS (0 = worsened, 1 = improved). Nagelkerke’s R2 = goodness of fit index (ranges from 0 to 1). A-D Odds ratio for each category of the variable Relationship with the patient: A= partner vs. other, B= parent vs. other, C= brother/sister vs. other, D= son/daughter vs. other. | | | | | |  |

| **Table S4** | | | | | |
| --- | --- | --- | --- | --- | --- |
| *Logistic regression models for grief change (dichotomized GS change variable as dependent variable)* | | | | | |
|  | | | | | |
|  | Independent variables | Nagelkerke’s  R^2^ | P value | Odds ratio  OR | OR Confidence interval |
| Univariate  models | Age | 0.032 | **.039** | **0.968** | **0.939 – 0.998** |
|  | Sex | 0.018 | .104 | 0.605 | 0.330 – 1.109 |
|  | Relationship with the patient | 0.034 | .366 | A – 3  B – 1.865  C – 6  D – 4 | 0.452 – 19.928  0.364 – 9.572  0.671 – 53.681  0.431 – 37.108 |
|  | Education (years) | 0.002 | .647 | 1.019 | 0.940 – 1.104 |
|  | Diff_BDI-II | 0.072 | **.001** | **2.843** | **1.496 – 5.402** |
|  | Diff_SCL-90 | 0.029 | **.040** | **1.906** | **1.029 – 3.530** |
|  | Diff_STAXI-2 ER/IN | 0.017 | .131 | 1.600 | 0.870 – 2.943 |
|  | Diff_STAXI-2 ER/OUT | 0.015 | .156 | 1.549 | 0.846 – 2.837 |
|  |  |  |  |  |  |
| Multiple model | Age  Diff_BDI-II  Diff_SCL-90 | 0.113 | **.043**  **.005**  .472 | **0.967**  **2.738**  1.290 | **0.937 – 0.999**  **1.352 – 5.545**  0.644 – 2.582 |

*Note.* Diff_BDI-II= Change T0-T1 in BDI-II (0= worsened, 1= improved); Diff_SCL-90= Change T0-T1 in SCL-90 (0= worsened, 1= improved); Diff_STAXI-2 ER/OUT= Change T0-T1 in STAXI ER OUT (0= worsened, 1= improved). Nagelkerke’s R^2^ = goodness of fit index (ranges from 0 to 1). A-D Odds ratio for each category of the variable Relationship with the patient: A= partner vs. other, B= parent vs. other, C= brother/sister vs. other, D= son/daughter vs. other.

| **Table S5** | | | | | | | | | | |
| --- | --- | --- | --- | --- | --- | --- | --- | --- | --- | --- |
| *Correlation Matrix* | | | | | | | | | | |
| Measure | 1 | 2 | 3 | 4 | 5 | 6 | 7 | 8 | 9 | 10 |
| 1. Age |  |  |  |  |  |  |  |  |  |  |
| 2. Education | -0.11 |  |  |  |  |  |  |  |  |  |
| 3. delta BAS | -0.15 | 0.05 |  |  |  |  |  |  |  |  |
| 4. delta GS | -0.22** | 0.06 | 0.50** |  |  |  |  |  |  |  |
| 5. delta BDI-II | -0.18* | 0.17 | 0.49** | 0.44** |  |  |  |  |  |  |
| 6. delta SCL-90 | -0.14 | 0.02 | 0.45** | 0.34** | 0.61** |  |  |  |  |  |
| 7. delta STAXI-2 ER/OUT | -0.04 | -0.06 | -0.09 | 0.09* | -0.01 | 0.16** |  |  |  |  |
| 8. delta STAXI-2 ER/IN | -0.02 | 0.12 | 0.13* | 0.22** | 0.17** | 0.19** | 0.24** |  |  |  |
| 9. delta FF | -0.17* | 0.06 | 0.34** | 0.27** | 0.22** | 0.25** | -0.11 | 0.20** |  |  |
| 10. delta TAS | -0.06 | -0.01 | 0.16* | 0.10* | 0.24** | 0.30** | 0.14* | 0.14* | 0.14* |  |
| *Note.* **p* < .05. ** *p* < .01. | | | | | | | | | | |
